# Bridging Gaps: A Comparative Approach to Managing Midline Diastema

**DOI:** 10.7759/cureus.28355

**Published:** 2022-08-24

**Authors:** Mrinal Nadgouda, Aditya Patel, Pradnya Nikhade, Manoj Chandak, Radhika Gupta

**Affiliations:** 1 Department of Conservative Dentistry and Endodontics, Sharad Pawar Dental College and Hospital, Datta Meghe Institute of Medical Sciences, Wardha, IND

**Keywords:** aesthetic buildup, composite resin, prefabricated strip crowns, midline diastema, sectional matrix

## Abstract

Diastema is a common finding in many populations, resulting in poor aesthetics and increasing self-consciousness when smiling. The diastema closure can be done conservatively without the use of braces by employing a variety of alternative treatment techniques, such as direct composite veneers, indirect composite veneers, porcelain laminate veneers, all-ceramic crowns, and metal-ceramic crowns. With the running trend amongst the youth of desiring faster aesthetic results in a stipulated timeframe, it has become a tedious job for the endodontist to satisfy these demands. In this time of crisis, a composite resin buildup offers a one-stop solution to all the vivid demands of the patients. This case report presents a less cumbersome procedure for the correction of midline diastemas by simple usage of stainless steel sectional matrix bands and anterior transparent crowns, which have proved beneficial for correcting these defects. To achieve an ideal smile and superior aesthetics, the usage of direct restorable composites onto the tooth surfaces has proven beneficial, providing the ultimate durability, pleasing aesthetics, and complete patient satisfaction.

## Introduction

Aesthetics has been a leading concern in today’s era amongst the younger generation. The increasing demand for tooth-coloured restorations has become a popular choice. Discrepancies in the anterior aesthetic zones are seen in the form of fracture, microdontia, mesiodens, Talon's cusp, and high frenal attachment leading to midline diastema. Amongst these aesthetic concerns, the management of midline diastema has gained popularity amongst the general public. A diastema is a distance or space between two or more adjacent teeth. A midline diastema is a space between the maxilla's or mandible's first incisors. Causes of midline diastema have been considered to be multifactorial, including thumb sucking, tongue thrusting, or lip sucking. Dental malformations such as proclination in the maxillary incisor, jaw discrepancy, and imperfect fusion of the dental septum can cause an unaesthetic smile, phonetic disorders, and interference in maintaining oral hygiene [1].

Various alternative treatment plans are available for the treatment of midline diastema, which include orthodontic appliances, restorative techniques, and prosthodontic rehabilitation. A carefully developed differential diagnosis allows the practitioner to choose the most effective treatment plan based on time constraints, and physical, physiological, and economic difficulties. Any of the following procedures can be used to conservatively close diastemas: direct composite veneers, indirect composite veneers, porcelain laminate veneers, all-ceramic crowns, and metal-ceramic crowns. Composite resin has now increasingly been used in the closure of midline diastema due to its resemblance with enamel and dentin in terms of its physical and chemical properties. The availability of various shades and opacities allows the patient to achieve an aesthetically pleasing smile [2]. Various corrective methods such as silicone index technique, ceramic crowns, laminates, and veneers can be used. With this overview, a few techniques for the closure of midline diastema have been incorporated into the two cases presented, namely the sectional matrix technique and prefabricated strip crowns. 

## Case presentation

Case 1: sectional matrix technique for diastema closure

A case of midline diastema closure was done where a 30-year-old male patient reported to the Department of Conservative Dentistry And Endodontics with a complaint of spacing in the upper front region of the jaw. His history revealed that he had the spacing from the time of eruption of permanent dentition and was reluctant to socialize among the public because of the same. On checking the cusp to fossa relationship, a class one type of Angle’s malocclusion was found present in the patient. No caries was detected in all four quadrants between tooth numbers one and seven. The labial frenum was in normal size and position.

**Figure 1:**
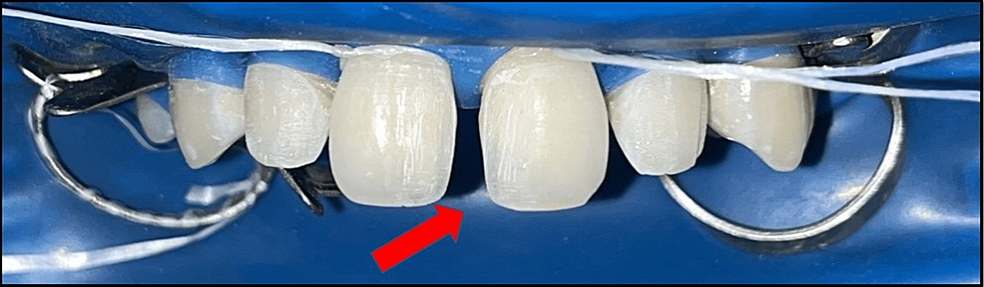
Teeth isolated using a rubber dam.

**Figure 2:**
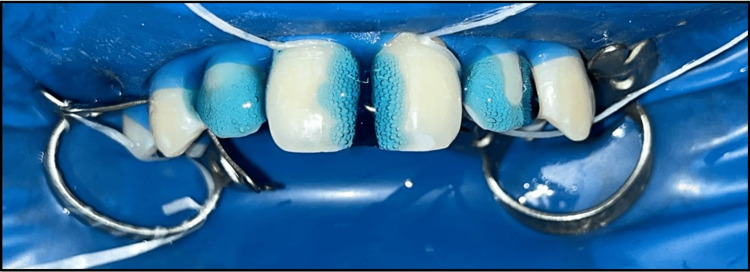
Etching done with 37% phosphoric acid on tooth concerning maxillary permanent right and left central incisor and maxillary permanent right and left lateral incisor.

**Figure 3:**
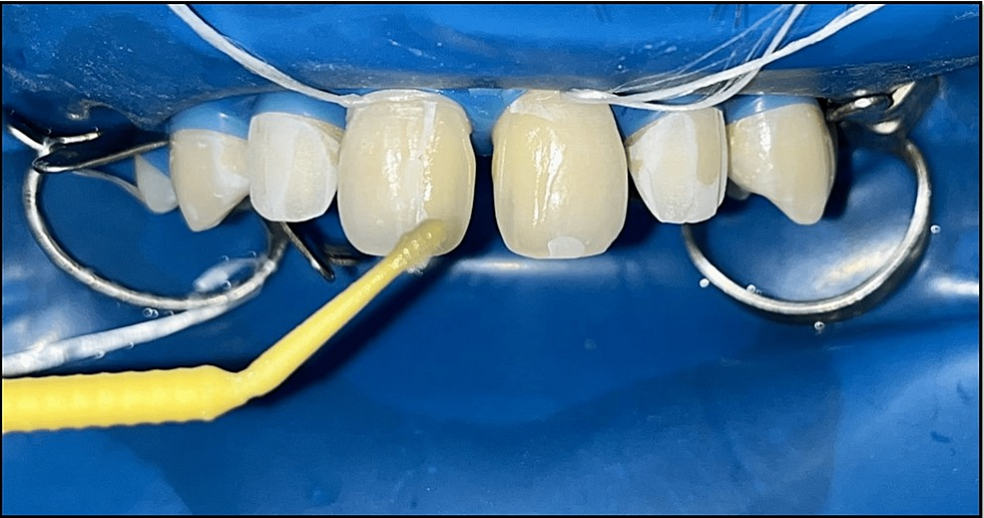
Bonding agent applied on tooth concerning maxillary permanent right and left central incisor and maxillary permanent right and left lateral incisor.

**Figure 4:**
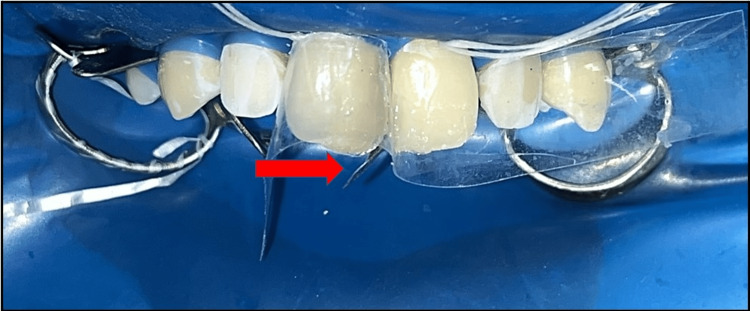
Mylar strip and sectional matrix used for closing the diastema between maxillary permanent right and left central incisor and maxillary permanent right and left lateral incisor.

Teeth were isolated following standardized isolation protocols using a rubber dam (Figure 1). Composite dots were placed on the tooth surfaces for appropriate shade to match the remaining natural dentition. The final shades selected were the A1 composite shade for the incisal buildup and A2 composite shade for the cervical buildup, selected from Spectrum® Universal Micro Hybrid Composite (Dentsply Sirona, Konstanz, Germany). A tapered fissure coarse diamond bur (TF-11) was taken, and a bevel was prepared on the incisal, middle, and cervical surfaces of the maxillary right permanent central incisor and maxillary left permanent central incisor. Etching of the teeth was done using 37% phosphoric acid for 20 seconds (Figure 2). Teeth were rinsed using water and air dried. A thin layer of bonding agent was applied, followed by air drying for uniform spread (Figure 3). The palatal shelf was built with the help of mylar strips and proximal walls were built using a sectional metal matrix band (Figure 4).

**Figure 5:**
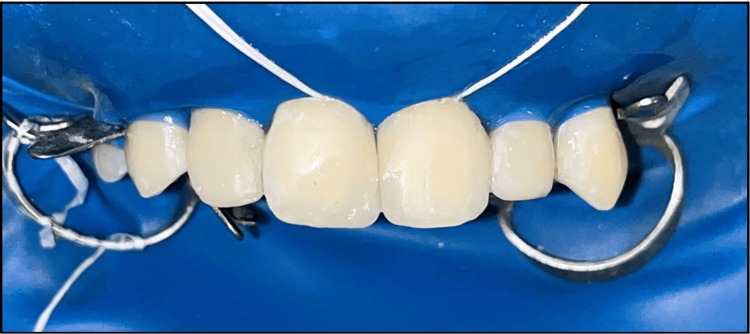
Extra fine tapered diamond bur used for gross finishing.

**Figure 6:**
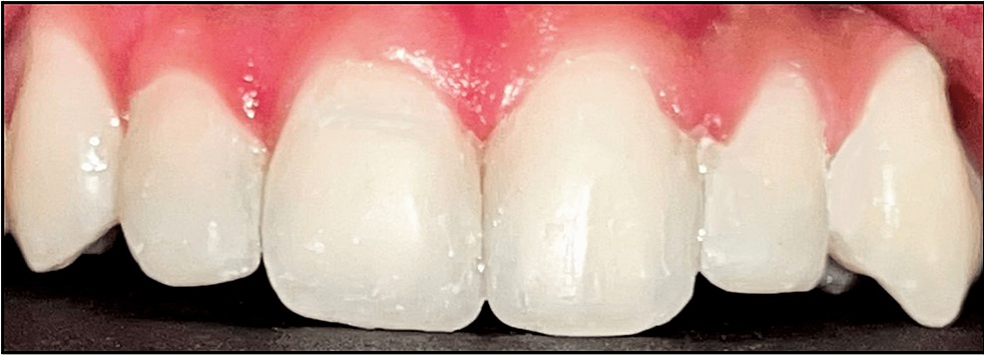
Final finishing and polishing.

Then composite resin was incrementally layered over the tooth surfaces and cured. An extra fine tapered diamond bur (TF-11) was used for the gross finishing of the composite (Figure 5). Final finishing and polishing were done with a series of abrasive disks (Figure 6). 

Case 2: prefabricated strip crowns for diastema closure

**Figure 7:**
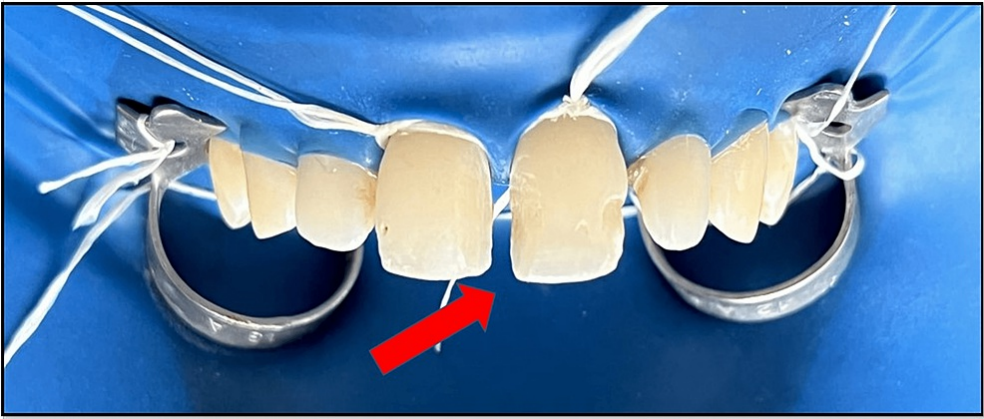
Teeth isolated using a rubber dam.

A novel, innovative, experimental approach for closing midline diastema was tried in a 27-year-old female patient who reported to the Department of Conservative Dentistry and Endodontics with the chief complaint of gaps between her teeth in the upper front jaw region. Spacing was present between teeth since early childhood, which was due to the eruption of permanent teeth and was not aesthetically appealing since then. She was, however, enthusiastic in opting for aesthetic corrections to close her spaces. History was recorded as per the previous cases. The patient displayed an Angle's class two bimaxillary malocclusion on checking the cusp to fossa relationship. No caries was detected in all four quadrants between tooth numbers one and seven. The labial frenum was in normal size and position. Teeth were isolated following standardized isolation protocols using a rubber dam (Figure 7).

**Figure 8:**
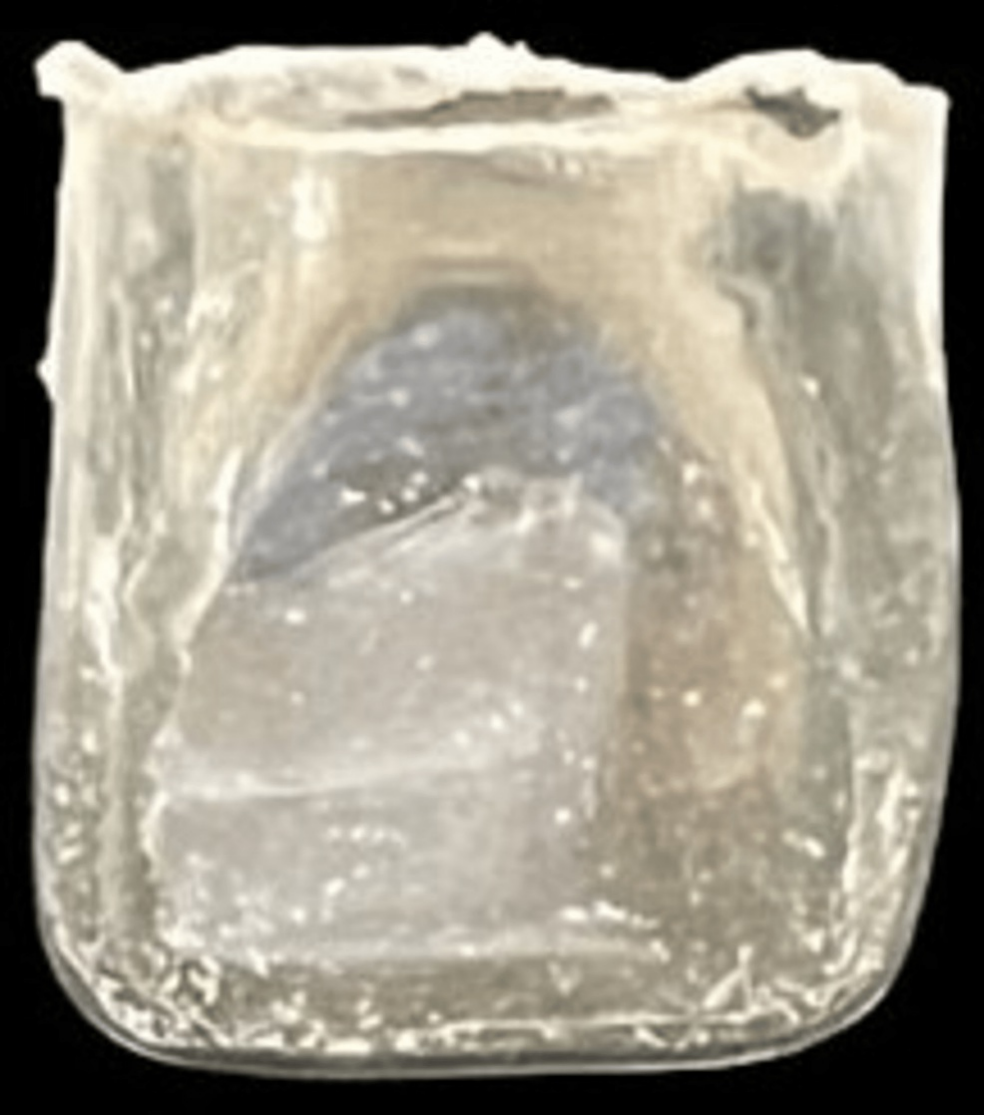
Tor Vm Anterior Transparent Crown (Tor Vm, Moscow, Russia).

**Figure 9:**
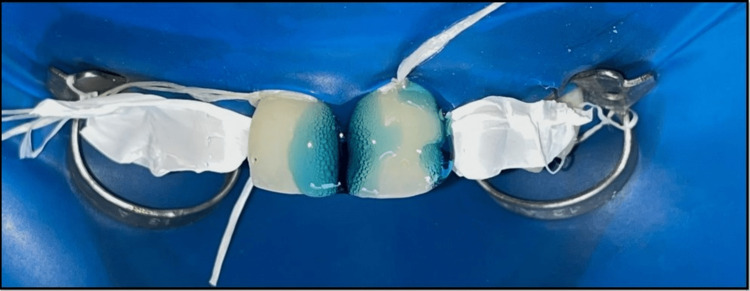
Etching done with 37% phosphoric acid on maxillary permanent right and left central incisor.

**Figure 10:**
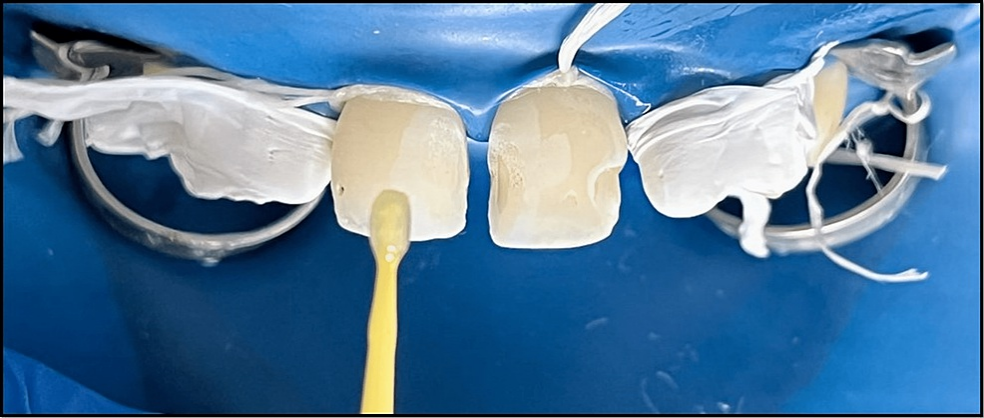
Bonding agent applied using a micro brush on tooth concerning maxillary permanent right and left central incisors.

**Figure 11:**
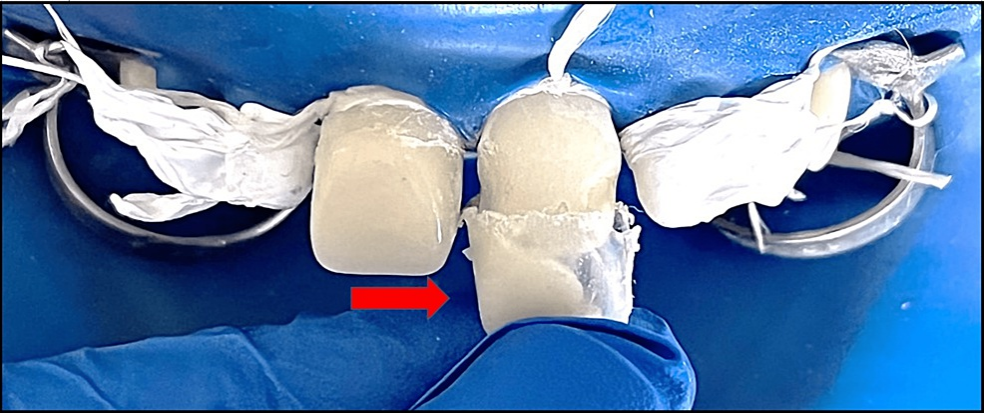
Composite increment placed within the strip crown and crown placed with light pressure at maxillary permanent right and left central incisor followed by the same technique applied with maxillary permanent right and left lateral incisors.

Composite dots were placed on the tooth surfaces for appropriate shade to match the remaining natural dentition. A2 composite shade was selected for both the incisal and the cervical region buildup from the available Spectrum® Universal Micro Hybrid Composite shade guide. Using a tapered fissure bur (TF-12), bevels were created on the maxillary right permanent central incisor and maxillary left permanent central incisor. A prefabricated maxillary central incisor strip crown (Tor Vm Anterior Transparent Crown; Tor Vm, Moscow, Russia) was selected, and its appropriate dimensions were matched according to the tooth size (Figure 8). Etching was done for 15 seconds and air dried (Figure 9) followed by the application of bonding agent and light curing for 20 seconds (Figure 10). Composite was incorporated within the crown, which was pressed onto the buccal and lingual tooth surface of the maxillary permanent right central incisor with light pressure. This caused the excess material to flow out of the strip crown (Figure 11).

**Figure 12:**
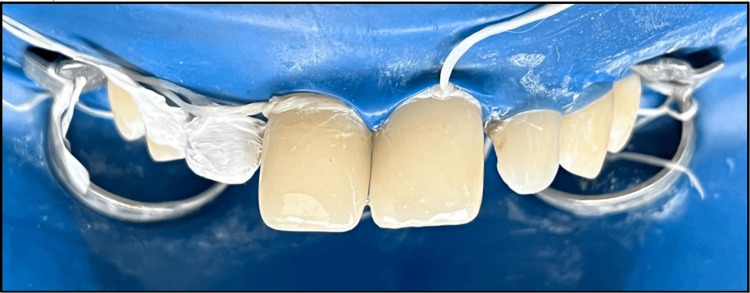
Extra fine tapered diamond bur used for gross finishing.

**Figure 13:**
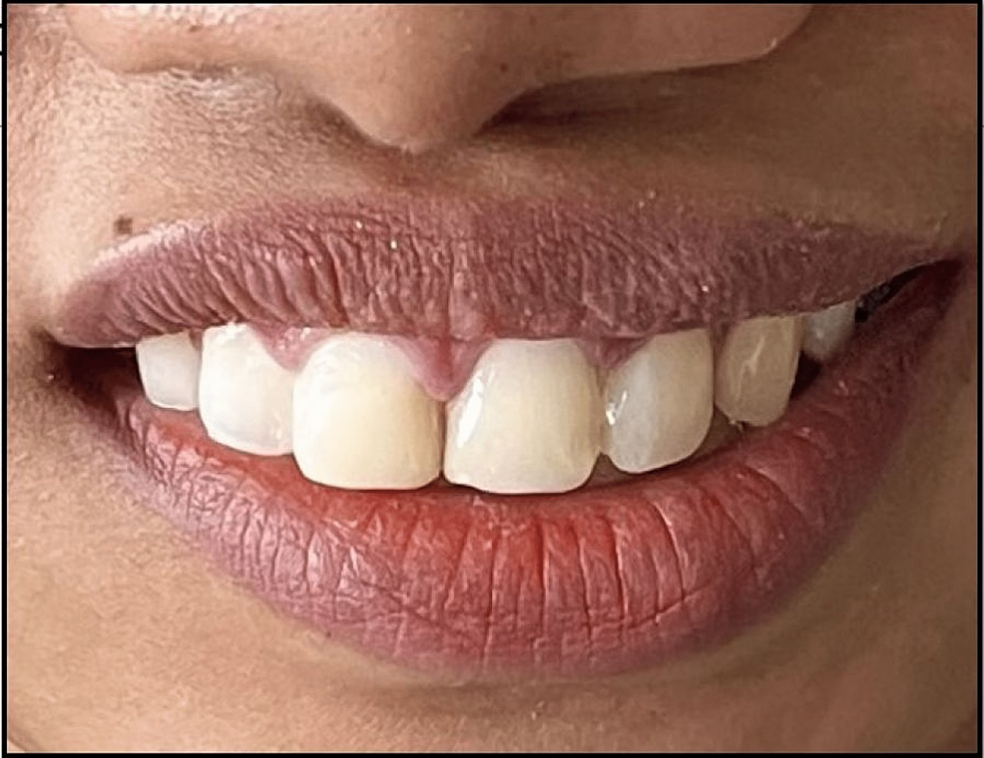
Final finishing and polishing.

Subsequently, light curing of the crown was carried out for a duration of 20 seconds. Following a complete set of the composite resin, the crown was cut from the distal aspect and removed to enhance easy separation of the crown. A similar procedure was repeated on the maxillary permanent left central incisor, and the crown was removed similarly. Spacing between maxillary permanent right central incisor, maxillary permanent left central incisor, and maxillary permanent left lateral incisor was also closed using strip crowns of shapes resembling the lateral incisors. An extra fine tapered diamond bur (TF-11) was used for the gross finishing of the composite (Figure 12). As for the remaining dentition, the final tooth contouring was done to replicate the anatomical form of the natural dentition. Final finishing and polishing were done with a series of abrasive disks (Figure 13).

## Discussion

The utilization of direct composite restorations for bridging gaps in cases of midline diastemas is known to all. Direct composites undoubtedly are the material of choice for anterior restorative procedure buildup. These resin-based composite restorations require a single visit appointment, avoid the laboratory fabrication time, and reduce the expenses involved in fabrication. They also have the advantage of not requiring wax-ups or preliminary model preparation, are soft on opposing dentition, and are simple to repair in fractures. This, however, seems impossible in cases of porcelain restorations that require an elaborate procedure for fabrication [3]. However, these restorations have some significant drawbacks, making case selection crucial. When compared to ceramics, composite restorations have inferior colour stability.

To replicate the exact emergence profile while maintaining the anatomical morphology of the reconstructed tooth, it is essential for the clinician that as described in the literature, appropriate finishing and polishing procedures should be made. This can be achieved with the use of a mylar strip to avoid the formation of open gingival embrasures [4]. Also, their inability to possess fracture toughness and bear compressive and shear strength render them unsuitable for usage in high-stress bearing occlusal areas [5]. One common practice to close midline diastema is to use a sectional metal matrix band. These bands are readily available for building missing walls, i.e., mesial, distal buccal, or palatal, in cases where the tooth has undergone a significant loss of tooth structure.

In the first case presented here, the sectional matrix band was first placed in the medial aspect of the right central incisor teeth in between the two central incisors to build up the deficient mesial walls. Packable microhybrid composite was incrementally placed in this region of specific interest and adapted onto the mesial wall of the central incisor tooth. This was then light cured to allow complete polymerization of the material. Metal matrices are extremely effective where significant diastemas (3-4mm) should be closed with direct composite resin restorations. In this situation, the matrices employed are polished stainless steel matrices meant for single use only. They pose no risk of epithelial damage when passively put into the sulcus, given that they are polished and made of soft metal. Before clinical usage, the manufacturers recommend steam autoclaving the matrices at 134 degrees Celsius for three minutes [1].

On the other hand, the second case presented here involves the utilization of pre-contoured anterior cellophane transparent crowns. They are, however, used for building tooth structures lost due to tooth fracture, possibly in cases of trauma to the anterior jaw regions. The advantages conferred by these crowns are that it is a quick, simple, and cost-effective method for aesthetic restoration of incisors and decreases the chair side time required for the building up of tooth in cases of young patients with a history of traumatic episodes. Added to this, they also minimize the final finishing and polishing time needed for achieving the final aesthetic demands of the patient. Disadvantages include tearing up of the crown during removal from the anterior region, over-expansion or outflow of the composite resins out of the required tooth surfaces, unnecessary handling problems, and unavailability of all sizes of teeth and shades for proper tooth contouring and shade matching.

The techniques mentioned in this case report are easy to perform, lucid, and cost-effective solutions for diastema closure. Ultimately, it is the clinician’s ability to close the diastema that plays a significant role in closing the gaps. The proper case selection, judgment, clinician’s skill, and expertise play a pivotal role in such case scenarios. For achieving successful and desired outcomes irrespective of the techniques used, the critical factor still remains to follow the standard isolation protocols judiciously. In these cases presented here, proper isolation of the operating field was maintained through a rubber dam sheet. This is due to two factors. The midline of the teeth should ideally correspond with the midline of the face, and while correcting midline diastema, it isn't easy to perceive the midline of the face with the rubber dam in place [6]. The finishing and polishing was preceded by gross reduction of the restoration using extra fine tapered composite finishing bur (TC-21EF) followed by polishing which was carried out by using Shofu Super-Snap kit (Shofu, Delhi, India). A follow-up of the above cases is of foremost importance to check for colour fading, microleakage, and thinning of the restoration, all of which hamper the longevity and quality of the restorations. Thus, a mandatory follow-up of patients must be envisaged to obtain maximum benefits from the usage of these techniques. 

## Conclusions

Orthodontic rehabilitation has been a treatment of choice for many years. Still, due to its extensive and long-term procedure, people are opting for more minimalistic approaches like composite restorative solutions. The cases presented here involve a few available treatment methodologies for correcting midline diastemas in clinical practice. They offer a lucid, easy-to-use, and cost-effective method in ultimately bridging the gaps of midline diastemas. This area in aesthetic dentistry is constantly evolving, and it can be expected that better results will soon be available by using advanced treatment protocols for the patient's benefit.
